# A Role for the Juxtamembrane Cytoplasm in the Molecular Dynamics of Focal Adhesions

**DOI:** 10.1371/journal.pone.0004304

**Published:** 2009-01-28

**Authors:** Haguy Wolfenson, Ariel Lubelski, Tamar Regev, Joseph Klafter, Yoav I. Henis, Benjamin Geiger

**Affiliations:** 1 Department of Neurobiology, George S. Wise Faculty of Life Sciences, Tel Aviv University, Tel Aviv, Israel; 2 School of Chemistry, Sackler Faculty of Exact Sciences, Tel Aviv University, Tel Aviv, Israel; 3 Freiburg Institute for Advanced Studies (FRIAS), University of Freiburg, Freiburg, Germany; 4 Department of Molecular Cell Biology, Weizmann Institute of Science, Rehovot, Israel; University of Birmingham, United Kingdom

## Abstract

Focal adhesions (FAs) are specialized membrane-associated multi-protein complexes that link the cell to the extracellular matrix and play crucial roles in cell-matrix sensing. Considerable information is available on the complex molecular composition of these sites, yet the regulation of FA dynamics is largely unknown. Based on a combination of FRAP studies in live cells, with *in silico* simulations and mathematical modeling, we show that the FA plaque proteins paxillin and vinculin exist in four dynamic states: an immobile FA-bound fraction, an FA-associated fraction undergoing exchange, a juxtamembrane fraction experiencing attenuated diffusion, and a fast-diffusing cytoplasmic pool. The juxtamembrane region surrounding FAs displays a gradient of FA plaque proteins with respect to both concentration and dynamics. Based on these findings, we propose a new model for the regulation of FA dynamics in which this juxtamembrane domain acts as an intermediary layer, enabling an efficient regulation of FA formation and reorganization.

## Introduction

Integrin-mediated cell adhesion to the extracellular matrix (ECM) occurs through specialized multi-molecular complexes termed focal adhesions (FAs) [1]–[4]. The mechanisms underlying the dynamic regulation of FA assembly and reorganization are critical to FA function in tissue scaffolding and cell signaling, thus affecting processes such as cell migration, wound repair and tissue morphogenesis [5]–[8], as well as survival, growth and differentiation [2], [7]–[9].

Thus far, information concerning the regulation of FA dynamics is scarce. Studies based on image correlation spectroscopy provided a measure of the coupling between adhesion components and actin [10], and fluorescent speckle microscopy was employed to explore interactions between F-actin and FA-associated molecules [11], revealing an apparent hierarchical flow of FA proteins and actin through FAs. Another recent study demonstrated that paxillin dynamics in FAs are regulated by FA assembly/disassembly, location in the cell and treadmilling [12]. These reports contributed to the understanding of the dynamic relationships between actin and FA proteins. In addition, several fluorescence recovery after photobleaching (FRAP) studies have addressed the dynamic properties of numerous FA proteins [13]–[22]. These studies, which indicated that FAs are molecularly dynamic sites, have mostly estimated a single FRAP halftime, without providing detailed mechanistic analyses of the processes involved.

To address this issue, we investigated the membrane association dynamics of fluorescence-tagged FA-associated proteins (paxillin, vinculin and β_3_-integrin) within and around FAs. To this end, we employed highly sensitive FRAP studies combined with *in silico* experiments and mathematical modeling to fit the data to fluorescence recovery by diffusion, exchange, or combination of both. Our results reveal the existence of several sub-domains, each characterized by distinct mechanisms controlling the dynamics of FA-associated proteins. Of particular importance is the novel notion that that there exists a juxtamembrane cytoplasmic region surrounding FAs, characterized by higher concentrations of FA proteins (*e.g.*, paxillin and vinculin), whose diffusion is attenuated in this environment. The dynamics in this area differ from the exchange-dominated dynamics of FA-bound molecules, and from the diffusion-based dynamics seen in the cytoplasm. These findings lead us to suggest a new model for the regulation of FA steady-state dynamics.

## Results

### FA plaque proteins display several dynamically distinct subpopulations

**Figure 1:**
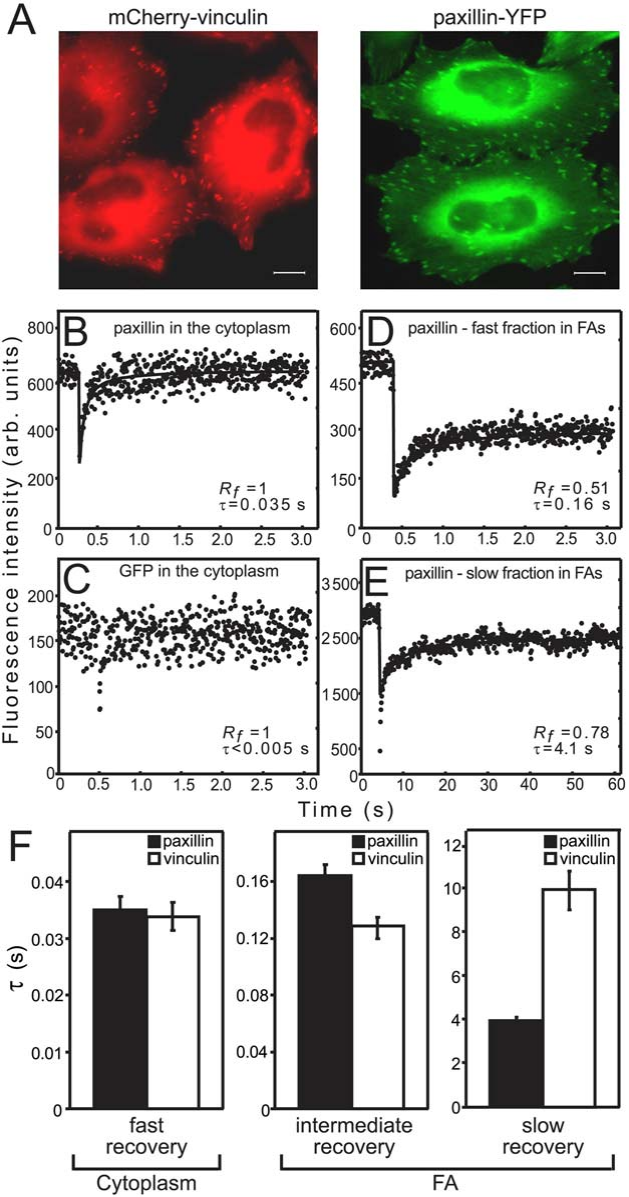
Typical curves demonstrating differences in paxillin and vinculin FRAP rates at different locations within the cell. (A) HeLa-JW cells expressing paxillin-YFP or mCherry-vinculin, plated on 20 μg/ml fibronectin-coated cover slips. (B–E) Typical FRAP curves of paxillin-YFP or free GFP in HeLa-JW cells. Cells were subjected to FRAP experiments 24–48 h after plating at 37°C, using a 63× objective (see Experimental Procedures). The temporal resolution (dwell time per channel) was 6 ms for the short time scale (3-s experiments), 120 ms for the long timescale FRAP studies on paxillin-YFP (60-s experiments), and 320 ms for the long timescale experiments on mCherry-vinculin (160-s experiments). Solid lines denote the best fit of a nonlinear regression analysis, fitting to a lateral diffusion process [24]; the resulting *τ* and *R_f_* values are shown. (B) FRAP of paxillin-YFP in the cytoplasm results in fast, complete recovery. (C) Free cytoplasmic GFP recovers instantaneously on the FRAP experimental timescale; therefore, fitting by non-linear regression was not possible. (D) FRAP of paxillin-YFP in FAs (3 s timescale). Lower *R_f_* and slower recovery relative to cytoplasmic paxillin were observed. (E) FRAP of paxillin-YFP in FAs (60 s timescale) resulted in higher *R_f_* and longer *τ*, as compared to (D). Note that fitting for *τ* ignored the first 6 points after the bleach, which correspond to the recovery phase shown in (D). (F) Average *τ* values for the subpopulations of paxillin and vinculin. Note the different timescales shown in each panel. Results represent the mean±SEM of 40–60 measurements, each conducted on a single FA within each cell and on different cells. In general, paxillin and vinculin displayed analogous patterns of dynamic subpopulations; the only major difference lay in the slow-recovering FA populations, where vinculin recovery was ∼2.5-fold slower.

To characterize the dynamic properties of plaque proteins at various cellular locations, we performed FRAP studies on HeLa-JW cells expressing paxillin-YFP or mCherry-vinculin (Figure 1A). These cells display relatively stable FAs that do not undergo structural reorganization on the FRAP timescale employed (up to 160 s). Combining a small illumination spot (focusing the Gaussian laser beam to 1.14 or 1.86 μm^2^) [23], a high-intensity bleaching beam (capable of achieving photobleaching in 2 ms), and a high-sensitivity photomultiplier, we were able to perform FRAP studies at high spatial and temporal resolution (4 ms), enabling discrimination between dynamic subpopulations of FA proteins at various locations both within and outside FAs.

We initially measured paxillin-YFP in the cytoplasm, focusing the laser beam at least 1 μm away from the ventral or dorsal membranes. These measurements yielded full recovery, with a short characteristic fluorescence recovery time *τ* (the time required to attain half of the recoverable fluorescence) (Figures 1B and 1F). Knowing the Gaussian radius of the beam (0.77 μm with a 63× oil objective), one can calculate the diffusion coefficient *D* [24], obtaining *D* = 4.0 μm^2^/s (see also Supporting Information). This result is close to the value of *D* determined by correlation spectroscopy for the cytoplasmic population of Lyn-GFP [25], and suggests relatively free cytoplasmic diffusion of paxillin-YFP. Yet, some restrictions on paxillin-YFP diffusion are likely to exist (see Discussion), since free cytoplasmic GFP (Figure 1C) exhibited even faster recovery (beyond the experimental timescale).

**Figure 2:**
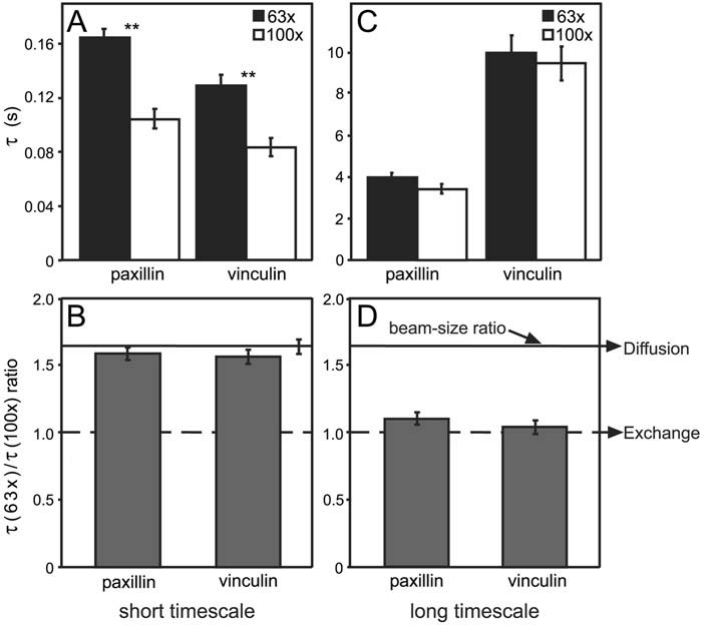
FRAP beam-size analysis reveals two different recovery modes inside FAs. FRAP experiments were conducted on HeLa-JW cells expressing paxillin-YFP or mCherry-vinculin, as described in Figure 1. Two beam sizes were generated using 63× and 100× objectives, and *τ* values were determined with each. The ratio between areas illuminated by the two beams was 1.63±0.03 (n = 59); this ratio is expected for FRAP by lateral diffusion, whereas a ratio of 1 is expected for recovery by exchange [23]. (A) *τ* values derived from FRAP experiments on a short timescale (3 s). For both paxillin and vinculin, the *τ*(63×) differed significantly from the *τ*(100×) value of the same protein (**, p = 2×10^−7^; Student's t-test). (B) *τ*(63×)/*τ*(100×) ratios derived from (A). The *τ* ratio for the 3 s measurements yielded 1.59 for paxillin and 1.58 for vinculin, close to the 1.63 ratio expected for lateral diffusion (solid line) (p = 0.24 and 0.15 for paxillin and vinculin, respectively; Student's t-test). (C) *τ* values from FRAP experiments on long timescales (60 or 160 s). For both proteins, the *τ*(63×) and *τ*(100×) values of the same protein were similar (p = 0.38; Student's t-test). (D) *τ*(63×)/*τ*(100×) ratios derived from (C). The *τ* ratios (1.09 for paxillin, 1.05 for vinculin) differed significantly from the 1.63 value for diffusion (p = 4*10^−23^ and 4*10^−25^ for paxillin and vinculin, respectively; Student's t-test). These values imply a major contribution of exchange to the recovery, as they are close to the ratio of 1 predicted for pure exchange (broken line). Bars in (A) and (C) represent means±SEM of 30–60 measurements. In (B) and (D), SEM of the *τ*(63×)/*τ*(100×) ratios were calculated using bootstrap analysis.

FRAP measurements taken on the same timescale (3 s), focusing the beam on the plasma membrane at FAs, resulted in *τ* values higher than those measured in the cytoplasm (Figures 1D and 1F), and a reduced mobile fraction (*R_f_*) of 0.49±0.02 (n = 48). Both effects can occur due to interactions of diffusing molecules with immobile entities [26], [27]. The particular type of effect depends on the FRAP timescale relative to the dissociation/association rates: stable interactions (*i.e*., long complex lifetimes relative to *τ*) would reduce *R_f_*, while transient interactions would increase *τ*, since each fluorescent molecule fluctuates between bound and unbound states during the measurement [26], [27]. Therefore, the higher *τ* of paxillin-YFP at FAs on the 3 s timescale indicates the existence of a population that interacts transiently with FAs. However, the reduction in *R_f_* indicates the existence of another subpopulation, with relatively stable interactions during the 3 s FRAP experiments. Since such interactions could become transient at longer times, we increased the FRAP timescale to 60 s, to allow sufficient time for additional paxillin-YFP molecules to dissociate. This would lead to an increase in *R_f_* and in *τ*. Indeed, under these conditions, *R_f_* increased to 0.81±0.02 (n = 61) (Figure 1E), accompanied by a 25-fold increase in *τ* (Figures 1E and 1F). A further increase in the FRAP timescale yielded no additional effects, suggesting that the remainder of the paxillin-YFP population (∼20%) is immobile for at least several minutes. Thus, apart from the fast-diffusing cytoplasmic population, the FA-associated paxillin-YFP consists of several subpopulations: one showing intermediate recovery on a 3 s timescale, another characterized by slow recovery, and a third which is immobile. In order to ensure that the slow recovery on the long FRAP timescale is not due tolateral diffusion within the FA itself, we employed the same beam size to bleach whole FAs by focusing the laser beam on small enough FAs that fitted entirely within the beam. Under these conditions, recovery of fluorescence cannot be from within the bleached FA. The recovery rates measured were similar to those in the standard experiments on larger FAs (not shown), validating that the recovery is from pools outside the FA, in keeping with the finding (Figure 2) that the recovery on the long timescale occurs mainly by exchange.

A similar pattern of dynamic subpopulations was observed for vinculin. Cytoplasmic vinculin displayed very fast recovery (Figure 1F); the fraction of mCherry-vinculin at FAs with an intermediate FRAP rate was somewhat faster than paxillin-YFP (Figure 1F), with a similar *R_f_* (0.50±0.01, n = 31); on the longer timescale, vinculin at FAs exhibited *R_f_* similar to paxillin (80%), albeit with 2.5-fold slower kinetics (Figure 1F).

In contrast to paxillin and vinculin, the transmembrane FA protein β_3_-integrin was essentially immobile in FAs on the timescale of our measurements (up to 5 min; data not shown), in accordance with previous reports [28]. This immobility, on a timescale close to the FA lifetime (10–30 min [29]), indicates that the recovery of the plaque proteins in FAs is not by lateral movement of large integrin-associated complexes within FAs, but rather by exchange with cytoplasmic plaque proteins.

### Different mechanisms govern the dynamics of fast- and slow-recovering subpopulations in FAs

FRAP beam-size analysis is a method recently developed by us [23], [30] to explore the membrane interactions of proteins that exchange between association with the plasma membrane and the cytoplasm. FRAP of intracellular proteins that interact with membrane-associated structures such as FAs can occur by diffusion and/or by exchange with cytoplasmic pools, and is therefore amenable to the same analysis [23], [30]. To characterize the recovery modes of paxillin and vinculin subpopulations at FA sites, we employed FRAP beam-size analysis [23], using 63× and 100× objectives to generate two different beam sizes, both small enough to fit within an FA (Figure 2). If FRAP occurs solely by diffusion, *τ* is proportional to the bleached area *τ_D_* = *ω* ^2^/4*D*, where *τ_D_* represents the characteristic diffusion time, and *ω* is the laser beam's Gaussian radius [24]. In this case, the ratio between the recovery times obtained with the two objectives, *τ*(63×)/*τ*(100×), should be 1.63 (the measured ratio between the illuminated areas). When FRAP occurs by exchange, *τ* reflects the chemical relaxation time, which is independent of the bleached area, *i.e*., *τ*(63×)/*τ*(100×) = 1 [23], [30]. Intermediate *τ* ratios suggest mixed recovery, in which the faster process plays a greater role [23], [30].

For both paxillin and vinculin, the interaction dynamics of the FA subpopulations that recover at intermediate (3 s timescale) or slow rates (60 or 160 s timescale) differ markedly (Figure 2). On the short timescale, *τ*(63×)/*τ*(100×) yielded 1.59 for paxillin and 1.58 for vinculin (Figures 2A and 2B), suggesting recovery mainly by diffusion. This enables the calculation of *D* from the *τ* values, yielding *D* = 0.9 and 1.15 μm^2^/s for paxillin and vinculin, respectively. These values are ∼4-fold lower than those obtained for the same proteins in the cytoplasm (Figures 1B and 1F), suggesting that they experience diffusion-attenuating interactions in the juxtamembrane cytoplasmic region above the FAs. The slow-recovering populations yielded *τ*(63×)/*τ*(100×) = 1.09 and 1.05 for paxillin and vinculin, respectively (Figures 2C and 2D); the fact that these values are significantly below 1.63 indicates a major contribution of exchange to the recovery. This situation precludes an accurate translation of *τ* to *D*; however, calculation of *D* and the exchange rate by fitting to models of FRAP by diffusion plus exchange (see below) yields *D* values similar to those measured for the fast-recovering populations (Supporting Information, Table S1).

**Figure 3:**
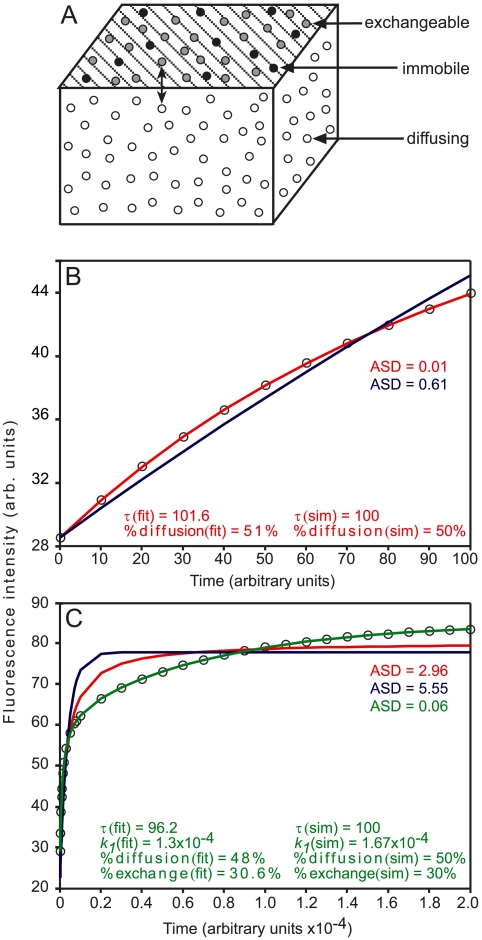
3D simulation of plaque protein dynamics and fitting to analytical expressions for different FRAP mechanisms. (A) Schematic representation of the model for plaque protein dynamics. The particles (molecules of FA plaque proteins) undergo fast 3D diffusion (random walk) in a cubic volume, and reversible binding to one of the volume boundaries (stripes). The bound molecules are assumed to be laterally immobile, to mimic FAs on the experimental timescale. A fraction of the bound molecules can, however, undergo exchange (slow, relative to the diffusion) with the free molecules. (B–C) FRAP simulations and fittings to different mechanisms. A CTRW algorithm [31], [32] was used to simulate FRAP experiments in a system modeled after paxillin and vinculin dynamics in FAs. The simulation parameters were chosen to resemble those of the FRAP experiments (Figures 1 and 2). Since the *R_f_* values of both proteins at FAs on the short timescale were around 0.50 (Figure 1D), 50% of the particles were denoted as undergoing 3D diffusion. Based on the increase in *R_f_* to ∼0.80 on the longer timescale (Figure 1E), the remaining 50% of the particles were divided, with 30% undergoing exchange, and 20% immobile. *τ* for diffusion (*τ_D_*) was introduced (in arbitrary units; au) as 100. To simulate a ∼60-fold slower exchange rate (see Figure 4), we introduced 1/*b* = *τ_D_*×60 = 100×60 au, *i.e.*, *b* = 1.67×10^−4^, where *b* represents the dissociation rate constant. (B) Short timescale. Under such conditions, the contribution of slow exchange was negligible, and it was sufficient to consider only the 3D diffusion. This was evident from the excellent fit of the data to the analytical expression for FRAP by lateral diffusion [24] (Eq. 14; red line). Fitting to exchange (Eq. 17; blue line) was much worse (Average Squared Deviation - ASD - values are shown). Fitting to two subpopulations (Eq. 18) did not improve the fit (data not shown). The parameters derived by fitting to lateral diffusion were similar to those introduced in the simulation [panel (B)]. Thus, the analytical expression for lateral diffusion [24] (Eq. 14) could be used to fit 3D diffusion. (C) Long timescale. At this timescale, the contribution of exchange becomes significant. The situation could be approximated by two subpopulations, one recovering by fast diffusion, and one by slow exchange (Eq. 18). Diffusion (red) or exchange (blue) alone did not fit. However, the analytical expression for two populations (Eq. 18; green) yielded a good fit. The fitted parameters were in agreement with those introduced in the simulation [Panel (C)].

To further support the notion of different recovery modes suggested by the FRAP beam-size analysis, we employed a complementary approach based on *in silico* simulations of a system modeled to resemble the dynamics of plaque proteins in FAs, as determined in the FRAP experiments (Figure 3). Based on the FRAP data (Figures 1 and 2), the system includes a population that recovers by diffusion in the cytoplasm (3D), and an FA-associated population that separates into two subpopulations, one undergoing exchange during the measurement, and the other which is immobile on this timescale (Figure 3A). The simulation data were generated using a constant time random walk (CTRW) algorithm [31], [32], and the results fitted to recovery by diffusion [24], exchange, or two subpopulations recovering by diffusion and exchange (see Supporting Information and Figure S1 for derivation of the analytical expressions). If the rate of diffusion in the cytoplasm is much faster than the rate of exchange (as is the case for paxillin and vinculin; Figure 2), the contribution of exchange is negligible at the short timescale required for diffusion, and the situation can be approximated by two populations, one recovering by diffusion and the other by exchange (see Eq. 18, Supporting Information). Although the diffusion in the cytoplasm is in 3D, the lateral diffusion equation [24] (Eq. 14) is still valid, since in FRAP experiments involving fluorescence collection from a restricted confocal plane, the 3D diffusion is projected into a 2D space (see Supporting Information). This finding is demonstrated by fitting the 3D simulated data on the short timescale, to the analytical expressions for 2D diffusion and/or exchange (Figure 3B). The lateral diffusion equation (Eq. 14) showed an excellent fit to the simulated data, yielding the correct *τ* value entered in the 3D simulation (Figure 3B). The analytical expression for two subpopulations undergoing diffusion and exchange (Eq. 18) did not improve the fit despite the additional degree of freedom, while exchange alone (Eq. 17) did not fit well at all (Figure 3B). Extending the timescale to cover the slower exchange process (Figure 3C) shows a good fit only to the analytical expression for two subpopulations undergoing diffusion and exchange (Eq. 18).

**Figure 4:**
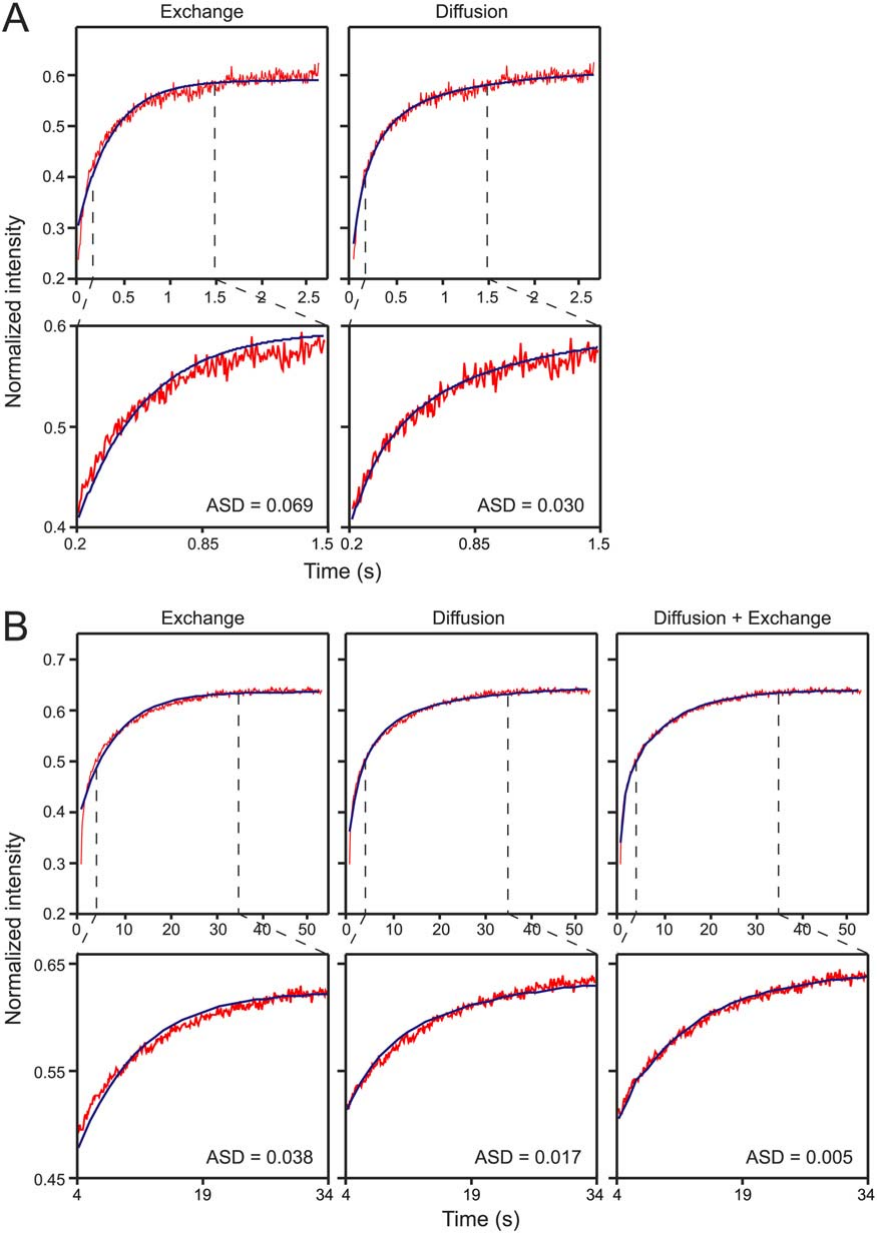
FRAP of paxillin in FAs fits recovery of two subpopulations by diffusion and exchange. FRAP data for paxillin-YFP in FAs (63× objective; experimental design as in Figure 1) was fitted to the analytical expressions for FRAP by lateral diffusion, by exchange, or by diffusion and exchange (two subpopulations; Figure 3). To improve the signal-to-noise ratio, 40–60 FRAP curves were averaged in each panel by summing up the intensities of each individual curve, starting from the bleach point for synchronization. To normalize the intensities, the pre-bleach level of each curve was given a value of 1. (A) FRAP on the 3 s timescale was well-fitted by diffusion. The diffusion equation [24] (Eq. 14, Supporting Information) yielded a good fit, while exchange (Eq. 17) was not well-fitted (see ASD values in lower panels). The *τ* value derived from this fit (0.15 s) was similar to that obtained by fitting each individual curve to lateral diffusion and averaging (Figure 1F; 0.16 s). The values obtained in the same manner for vinculin are depicted in Table S1. (B) FRAP of the slow-recovering fraction (60 s timescale). The fit obtained for the combination of diffusion and exchange (two subpopulations; Eq. 18) was better than the fit for each mechanism separately (see ASD values in lower panels). The fit yields *τ* = 0.15 s, similar to the 0.16 s value obtained for the fast-diffusing population, and *b* = 0.11 s^−1^ (*i.e.*, 1/*b* = 9.09 s). Due to the much slower exchange, the process can be approximated as one subpopulation recovering by diffusion, and the other by slow exchange (see Results).

We then proceeded to fit the experimental FRAP data to the different models (Figure 4). In FAs, the fraction that recovers on the short timescale (3 s) did not fit exchange, but was well-fitted by pure diffusion (Figure 4A). The fit was not significantly improved by combining diffusion and exchange (data not shown). The slow-recovering fraction (60 s timescale) was best fitted by the analytical expression for two subpopulations undergoing diffusion and exchange (Figure 4B). Similar results (not shown) were obtained for vinculin. These findings are in accord with the FRAP beam-size analysis.

### FRAP dynamics of plaque proteins differ at the proximal and distal FA ends

The “proximal end” of an FA (the edge pointing towards the attached actin bundle) and the “distal end” often display distinct assembly/disassembly kinetics [33]. Thus, FA “migration” can occur due to treadmilling, manifested by extension at the proximal end and dissociation at the distal one. To examine whether this asymmetry is reflected in paxillin and vinculin dynamics, we performed FRAP measurements at the two FA ends. Relatively long adhesions (>5 μm) were chosen to discriminate between them.

**Figure 5:**
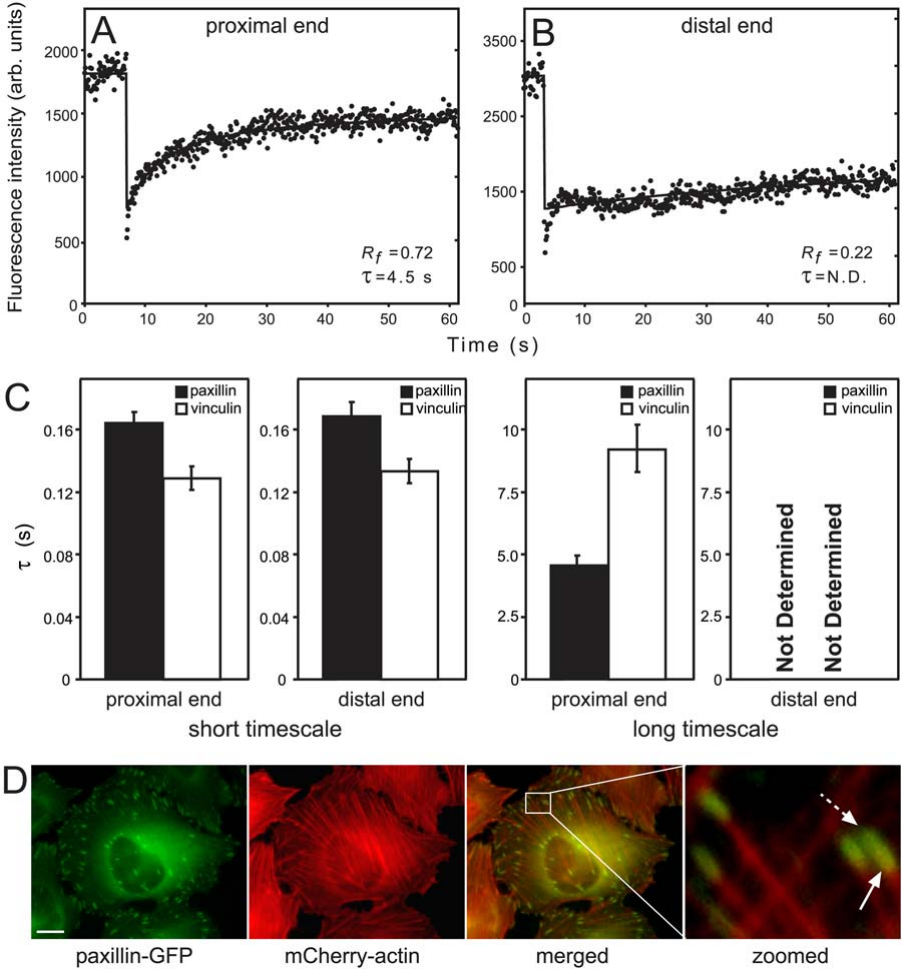
Paxillin and vinculin display different dynamics at the FA proximal and distal ends. FRAP experiments were carried out as described in Figure 1, focusing the beam on the two FA ends. (A) A typical FRAP curve of paxillin-YFP at the proximal FA end (60 s timescale). This curve is similar to curves obtained in the middle of smaller FAs on the same timescale (Figure 1E). (B) A typical curve at the distal FA end (60 s timescale). Fast recovery by diffusion exists, but the ensuing exchange is very slow, preventing determination of *τ*. To eliminate the contribution of the fast recovery, the first 6 points after the bleach were ignored in the fitting. (C) Average *τ* values of paxillin-YFP and mCherry-vinculin at the two FA ends. On the short timescale (left panels), which represents 3D diffusion (Figures 1, 2 and 4), the *τ* values were similar at both ends, resembling those at the centers of smaller FAs. On the long timescale (right panels), there were marked differences between the two: at the proximal end, significant recovery was observed at rates resembling those at smaller adhesions, while at the distal end, recovery was too slow to be measured. (D) Paxillin co-localizes with actin at the FA proximal end. HeLa-JW cells co-expressing paxillin-YFP and mCherry-actin were visualized by fluorescence microscopy (see Experimental Procedures). Co-localization was visible at the proximal edge (solid arrow), but not at the distal edge (dashed arrow). Scale bar: 10 μm.

On the short timescale, we found no differences between the dynamics at the two ends (Figure 5C). Furthermore, diffusion remained the major recovery mechanism, as indicated by beam-size analysis (data not shown). However, on the longer timescale (60 s for paxillin, 160 s for vinculin), FRAP revealed marked differences in dynamics at the proximal and distal ends. At the former, FRAP rates and *R_f_* values for paxillin and vinculin were similar to those measured at the centers of smaller adhesions (compare Figures 1E and 1F with Figures 5A and 5C). Beam-size analysis suggested that exchange remained the major FRAP mechanism. At the distal end, the recovery rate was extremely slow, enabling extraction of only *R_f_* (∼25%) (Figures 5B and 5C). Thus, the dynamics of the slow-recovering population depend on the location within FAs, with faster exchange occurring at the proximal end. The distribution of exchange rates correlates with the localization of actin stress fibers tips in FAs [34], which is lower at the distal end (Figure 5D). The correlation with actin density is in line with theoretical models proposing that the force exerted by the actomyosin machinery regulates FA dynamics [35], [36] (see Discussion).

### Variable dynamics of FA plaque proteins and β_3_-integrin in the vicinity of FAs

**Figure 6:**
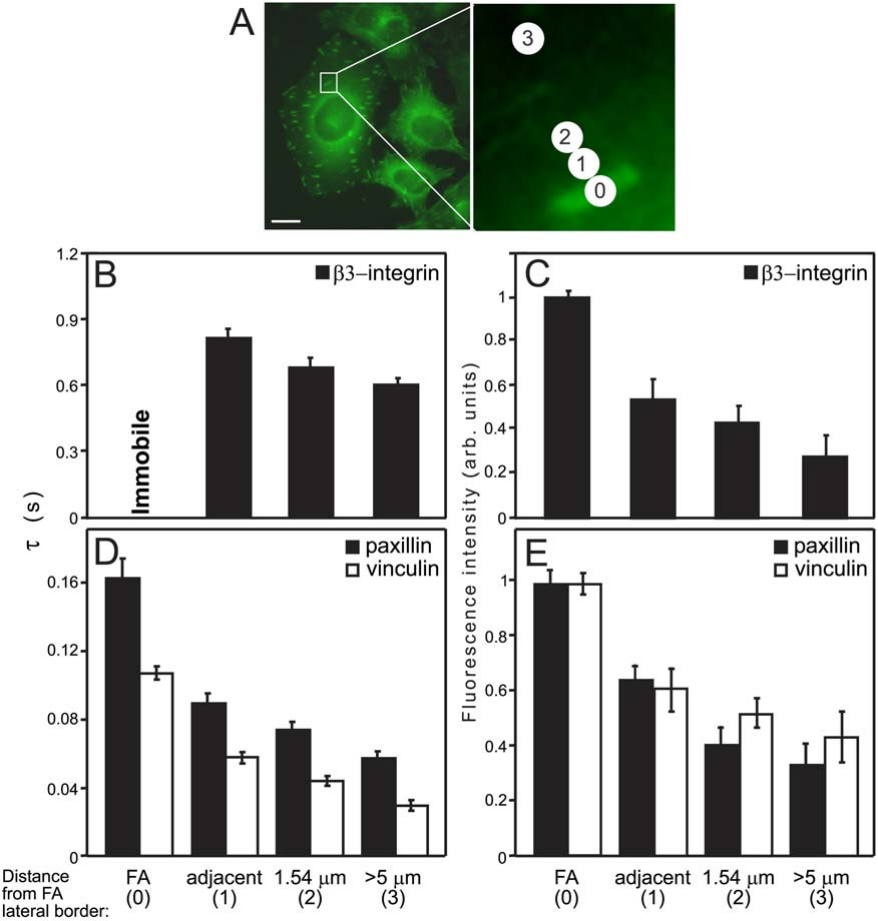
The rate of paxillin recovery outside FAs changes with distance from the FA edge. FRAP experiments were conducted as described in Figure 1. (A) Typical HeLa-JW cells expressing paxillin-YFP are shown, demonstrating clear, highlighted FAs with large differences in fluorescence intensity between adhesion and non-adhesion areas. Scale bar: 10 μm. FRAP experiments were performed at defined distances from the FA edge (right panel; scale bar: 1 μm). The numbers represent the position of the bleached regions relative to the FA. The locations of the bleached regions are defined as follows: 0 – inside the FAs; 1 – immediately adjacent to the FA edge; 2 – one beam diameter (1.54 μm) from the FA edge; and 3 – regions >5 μm from the FA edge. (B) Average τ values at the various locations for GFP-β_3_-integrin. While β_3_-integrin was virtually immobile at FAs, it was mobile outside the FA region, and displayed a gradient of recovery rates. (C) Relative average fluorescence intensities of GFP-β_3_-integrin at the various locations. Fluorescence intensities were quantified by using the FRAP instrumentation under non-bleaching conditions. The intensity of GFP-β_3_-integrin fluorescence inside FAs (location 0) was normalized to 1. (D) Average *τ* values at the various locations of paxillin-YFP and mCherry-vinculin. A gradient of recovery rates was observed for both paxillin and vinculin as a function of the distance from the FA edge. (E) Relative average fluorescence intensities of paxillin-YFP and mCherry-vinculin at the various locations. The respective intensities of each protein at location 0 were normalized to 1.

To examine whether the FA edge defines a sharp boundary for FA protein dynamics, we performed FRAP measurements of paxillin-YFP, mCherry-vinculin, and GFP-β_3_-integrin at varying distances from this edge, moving sideways within the plane of the ventral membrane in increments of 1.54 μm, equivalent to the laser beam diameter (Figure 6A). Although these proteins are concentrated in FAs, they are also present at lower densities outside FAs (Figures 6C and 6E). For β_3_-integrin, a dramatic change from immobility on our 1–3 minute timescale to high apparent mobility (*R_f_* = 0.85) was observed in regions immediately adjacent to the FAs (region 1; Figure 6B); calculation of *D* from *τ* yields *D* = 0.18 μm^2^/s. A gradual decrease in *τ* (higher *D* values) was found as distances from the FA edge increased (*D* = 0.20 and 0.25 μm^2^/s at distances of 1.54 μm and >5 μm from the edge, respectively).

Similar experiments with paxillin-YFP and mCherry-vinculin demonstrated that at all locations outside FAs, these proteins exhibited complete recovery (*R_f_* = 1) on the short timescale (3 s), suggesting the absence of the slow-recovering subpopulation observed in FAs. Beam-size analysis (Supporting Information, Figure S2) indicated that adjacent to the edge or one beam diameter away, FRAP of paxillin and vinculin occurred by an apparent mixture of diffusion and exchange. The transition from recovery by diffusion in FAs on the 3 s timescale (Figures 2 and 4) to FRAP with the increasing contribution of exchange (near FAs), suggests a faster exchange rate relative to diffusion in the latter case [23], as validated by fitting to recovery by diffusion and exchange (Supporting Information, Table S1). The faster recoveries of paxillin and vinculin near FAs, as compared to values within FAs (Figure 6D) suggest fewer binding sites and/or weaker transient binding (exchange) to membrane-associated protein complexes, resulting in less attenuation. These interactions appear to generate a gradient that decreases with distance from the FA. Indeed, at large distances from the FA (>5 μm), the mobility-restricting interactions fade away, leading to *τ* values resembling those observed in the cytoplasm (Figure 6D), and to recovery by diffusion (as in the cytoplasm; Table S1).

## Discussion

**Figure 7:**
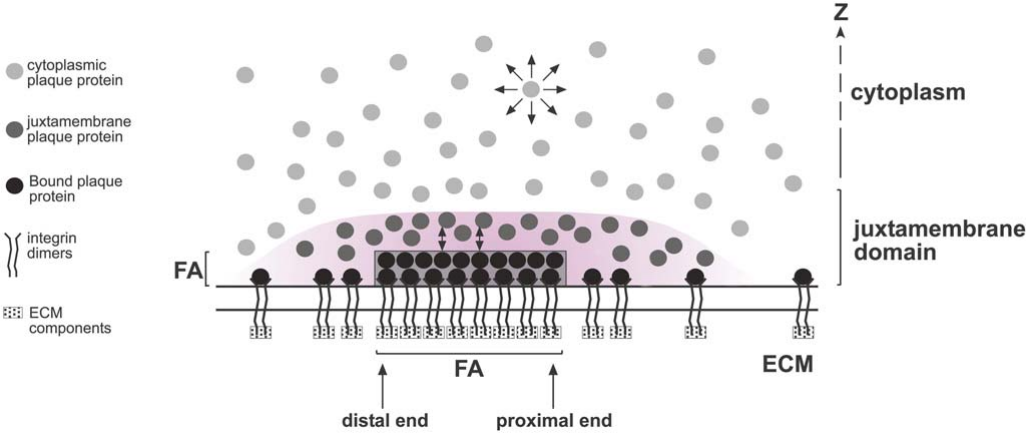
A schematic representation of the three spatial domains defined by FA protein dynamics. The results presented in this article point to the presence of three spatial domains within and around FAs, with distinct molecular dynamics: (i) the cytoplasm, characterized by fast-diffusing FA plaque proteins; (ii) the juxtamembrane region surrounding FAs, which extends into the z-axis as well as laterally (shaded area), where the plaque proteins display attenuated diffusion; (iii) the FA domain, containing two subpopulations of molecules, one associated with the FA surface and undergoing exchange (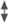), and an immobile, FA-bound subpopulation. The model is not drawn to scale for demonstrative purposes, and specifically the distance along the z axis is exaggerated in order to clearly depict molecules with a higher local concentration in this region. The juxtamembrane zone (shaded area) is actually adjacent (just above) the FAs, and has a gradient nature that decreases gradually with the distance from the FA both in the XY plane and in the z direction (see text). Depending on the polar density of actin stress fiber tips in FAs, the plaque proteins at the two FA ends (proximal, high actin fiber density; distal, low density) display different exchange rates. We propose that the density of integrins, which is higher at the FA region but is also not negligible outside FAs, attracts FA plaque proteins by both direct and indirect binding, leading to a density gradient of FA plaque proteins, as well as to distinct domains and dynamic populations.

In the present study, we demonstrate that FA plaque proteins are characterized by four distinct dynamic populations, which define three spatial domains (Figure 7). The interchanges and cross-talk among these domains can play critical roles in regulating FA stability and matrix adhesion. The three domains include: (i) the cytoplasm (>1 μm deep), where paxillin and vinculin display very rapid FRAP rates, though slower than those of free GFP (Figure 1); (ii) the juxtamembrane region surrounding FAs (50% of the FA-associated molecules), where the plaque proteins display attenuated diffusion at "intermediate" rates (*τ* = 0.12–0.16 s); and (iii) the FA domain, consisting of two subpopulations, one (30% of the molecules) that undergoes slow exchange (4–9 s; Figures 1, 2 and 4), and another (20%) which is essentially immobile on the experimental timescale, representing molecules stably associated with FAs.

The diffusion of paxillin and vinculin in the cytoplasm occurs at somewhat slower rates as compared to free GFP (Figure 1). This mild retardation is most likely due to their transient association with slower-diffusing or immobile proteins/structures, since the size of paxillin-YFP (93 KDa) or mCherry-vinculin (144 KDa) is still smaller than the size reported to result in steric constraints on cytoplasmic protein diffusion [37], [38].

The combination of FRAP beam-size analysis (Figure 2) and mathematical modeling (fitting to analytical expressions for diffusion, exchange or both in combination; Figure 4) demonstrates a two-step fluorescence recovery mechanism at FAs, composed of relatively fast diffusion (intermediate recovery rate on the 3 s timescale), followed by slow exchange. Therefore, FRAP measurements on the short timescale, where the contribution of slow exchange is negligible, fit recovery by diffusion (Figures 2 and 4). On a longer timescale (60–160 s) diffusion is completed during the initial phase of the recovery, and a major contribution is provided by exchange. Earlier studies on two-step recovery [22], [39] assumed a *D* value, and fitted only the exchange parameters. Here we show that when the rates of the two processes are well-separated, it is possible to approximate the data as two populations, one recovering by relatively fast diffusion, and the other by exchange (Figure 3). Fitting to the analytical expression of this model (Eq. 18, Supporting Information) enables derivation of the dynamic parameters characterizing each process (Figure 4 and Table S1).

To explain the attenuated (but still rather fast) diffusion of paxillin and vinculin around FAs, we propose a novel "juxtamembrane domain" or zone (Figure 7). The molecules within this domain are cytoplasmic, and show recovery by diffusion (Figures 2 and 4), albeit their diffusion rate is reduced relative to the remote cytoplasmic population. The attenuated diffusion rates of FA plaque proteins in this domain may be attributed to transient interactions, either among themselves, with the membrane cytoskeleton, or with other structures. Such transient interactions are capable of increasing the local density of the plaque proteins in the juxtamembrane region, provided that the target proteins (*e.g.*, integrins) that bind them are concentrated in FAs (Figure 7). This juxtamembrane domain may act as an intermediary between the fast-diffusing cytoplasmic pool and the FA-associated population, able to exchange with both. This could be advantageous for the fast regulation of FA formation and reorganization, particularly for the FA stress response [40]. It was shown that locally applied rapid stress induces FA elongation and increase in area, accompanied by recruitment of new molecules, within approximately one minute [40]. Such a quick response may be facilitated by the juxtamembrane domain, where the immediate availability of FA plaque proteins would eliminate the need to recruit them from the remote cytoplasmic pool. Notably, Figure 6 demonstrates that the juxtamembrane domain also extends to the sides of the FAs. In areas adjacent to the sharp borders of the FA (locations 1 and 2 in Figure 6), paxillin and vinculin displayed unique dynamic properties characterized by a gradient of recovery rates, with a mixed contribution of diffusion and exchange. The exchange rates at these locations were considerably higher than at FAs (*b*, the dissociation rate constant, ranges from 2 to 10 s^−1^ for both proteins; Table S1). However, at distances >5 μm from FAs, the dynamic properties were similar to those of the cytoplasmic population (*i.e.*, fast recovery by diffusion). A parallel gradient (reduced density and faster diffusion as the distances from FAs increased), appears to exist for β_3_-integrins as well (Figure 6C), suggesting that transient association with β_3_-integrins underlies the gradient of recovery rates observed for paxillin and vinculin, thus extending the juxtamembrane domain laterally.

We therefore propose a structural-dynamic model compatible with these results (Figure 7). In this model, integrins immobilized by binding to ECM components in FAs [41] provide a focal point for recruitment of FA plaque proteins. Outside FAs, integrins display significant mobility, while within FAs (Figure 6B) they are nearly immobile relative to the FA lifespan (minutes or more), suggesting very stable interactions with the ECM. Plaque proteins at the FA site interact locally with the cytoplasmic faces of the integrins, of which ∼20% are immobile (*i.e*., tightly bound to immobile targets). This may reflect heterogeneity in their binding sites on integrins, since multiple sites enabling direct or indirect binding can result in a variety of dissociation rates [3]. The existence of a spectrum of exchange rates is exemplified by the differing dissociation rate constants of the different plaque proteins (*e.g*., *b* = 0.11 s^−1^ for paxillin, and *b* = 0.025 s^−1^ for vinculin).

The dynamics of FA plaque proteins are also affected by their location within the FA itself. We detected differences between the exchange rates at the FA proximal and distal regions. At the proximal end, paxillin and vinculin display much faster exchange rates than at the distal end (Figure 5). Several factors could contribute to this behavior, taking into consideration the polarity of FAs, which is mainly affected by mechanical forces exerted *via* the attached actin stress fibers. This polarity is also manifested in the varying densities of phospho-paxillin, which affect FA assembly, turnover and stability [42]. As suggested by the co-localization of actin and paxillin within the FA, the forces exerted by actin stress fibers may differ at either end of the adhesion, resulting in variations in the exchange dynamics of FA plaque proteins (Figure 7).

In the current study, we examined FAs at steady-state, yet our results may also shed new light on two models previously constructed to explain the regulation of FA size [35], [36]. Both models theorize different flux rates of plaque proteins at the proximal and distal ends of FAs, a prediction corroborated by our observation of different exchange rates at these locations. The two models also suggest that at steady-state, plaque proteins from the proximal end should move laterally toward the distal end. However, we found no evidence for such a mechanism on the timescale of our measurements, since lateral diffusion within FAs did not contribute significantly to the recovery (see Results). An additional feature not included in these models is our novel finding of the existence of a juxtamembrane domain surrounding the adhesion (Figure 7), which may have important implications for FA dynamics. The availability of plaque proteins in the area surrounding the adhesion enables the treatment of the process in these models as kinetically limited (*e.g*., involving the dissociation rate of plaque proteins), rather than diffusion-limited.

We propose that juxtamembrane domains may be relevant not only to FAs, but also to other membrane-associated complexes. Signaling initiated by transmembrane growth factor receptors, for example, requires the binding of various components of the “signaling cascade” to the activated membrane receptors. Future challenges include defining the parameters that regulate the structure and function of the FA juxtamembrane domain, and directly testing the existence of and the roles played by juxtamembrane domains in other systems, such as clusters of growth factor receptors (*e.g.*, EGF receptor) or cell-cell adhesions.

## Materials and Methods

### Reagents and plasmids

Hanks Balanced Salt Solution (HBSS), phosphate buffered saline (PBS) and fibronectin were supplied by Sigma-Aldrich. Fugene transfection reagent was purchased from Roche.

### Cell Lines and transfections

The HeLa-JW and REF52 cell lines expressing either YFP-tagged paxillin or GFP-tagged β_3_-integrin were previously described [42], [43]. For experiments involving transient expression (mCherry-vinculin), cells were transfected with Fugene 6 (Roche). All cell lines were cultured in Dulbecco's Modified Eagle's Medium (DMEM) supplemented with 10% fetal calf serum (FCS), 100 U/ml penicillin, 100 μg/ml streptomycin, and 4 mM glutamine. All cell culture components were provided by Biological Industries, Beit Haemek, Israel.

### Fluorescence recovery after photobleaching (FRAP)

FRAP studies were conducted on live cells expressing the various fluorescence-tagged FA-related proteins. The cells were taken for FRAP experiments 24–48 h after being plated on glass cover slips coated with 20 μg/ml fibronectin. Measurements were taken in HBSS supplemented with 20 mM Hepes, pH 7.2, at 37°C. An argon ion laser beam (Innova 70C; Coherent) was focused through a fluorescence microscope (AxioImager D.1; Carl Zeiss MicroImaging, Inc.) to Gaussian spots of 0.60±0.01 (plan-apochromat 100×/1.4 NA oil immersion objective), 0.77±0.01 μm (plan-apochromat 63×/1.4 NA oil immersion objective), or 1.17±0.02 μm (C-apochromat 40x/1.2 NA oil immersion objective); experiments were conducted with each beam size [beam-size analysis [23], [44]]. The ratio between the illuminated areas was 2.29±0.02 (n = 39) using the 63× and 40× lenses, and 1.63±0.02 (n = 59) using the 100× and 63× lenses. After a brief measurement at monitoring intensity (488/528 nm, 1 μW), a 5 mW pulse (2–10 ms) was used to bleach 50–75% of the fluorescence in the spot. The time course of the fluorescence recovery was tracked by the attenuated monitoring beam. The apparent characteristic fluorescence recovery time *τ* and the mobile fraction *R_f_* were extracted from the FRAP curves by nonlinear regression analysis, fitting to a lateral diffusion process [24] or exchange process.

### Co-localization of paxillin and actin by fluorescence microscopy

HeLa-JW cells stably expressing paxillin-YFP were plated on 20 μg/ml fibronectin-coated cover slips. One day after plating, cells were transfected with 1 μg of a plasmid encoding mCherry-actin. After 24 h, cells were fixed with 4% paraformaldehyde, mounted with gel/mount containing anti-fading agents (Biomeda, Foster City, CA, USA) and visualized using a Zeiss Axio Imager D.1 fluorescence microscope with a 63×/1.4 NA objective. Fluorescence images were recorded using OED capture software with a CoolSNAP HQ-M CCD camera (Photometrics).

### Mathematical modeling

Detailed descriptions of the models and simulations, as well as derivations of the various analytical expressions, are presented in the Supporting Information.

### Statistical Analysis

Calculations of SEM for the beam-size ratios and the *τ* ratios in Figures 2 and S2 were performed using bootstrap analysis, a preferred method for ratio estimation [45]. The *τ* values from the FRAP experiments using the 63× and 100× lenses were resampled with replacement using Excel, and average values from each group of resampled data were extracted. For each lens, 100 average samples were generated in this way, followed by division of the 63× resampled data by the 100× resampled data. The group of 100 ratio values was then analyzed, using SPSS for average and SEM values. Estimation of the goodness-of-fit of the FRAP data to the various analytical expressions (Figures 4 and 5) was performed by calculating the Average Squared Deviation (ASD) value of each fit. The squared deviations between the calculated points (from the fit) and the simulated/experimental data were summed up, and then divided by the number of calculated points to obtain the ASD value.

## Supporting Information

Figure S1 1st figure for the Supporting Information
(0.84 MB EPS)

Figure S2 2nd figure for the Supporting Information
(0.60 MB EPS)

Table S1 1st table for the Supporting Information
(0.09 MB PDF)
