# Correction: A Role for the Juxtamembrane Cytoplasm in the Molecular Dynamics of Focal Adhesions

**DOI:** 10.1371/annotation/ce274a43-a284-4839-9791-59e4b2563c9c

**Published:** 2009-03-04

**Authors:** Haguy Wolfenson, Ariel Lubelski, Tamar Regev, Joseph Klafter, Yoav I. Henis, Benjamin Geiger

The Supporting Information text file does not appear. It should be cited as "Text S1", with a caption "Mathematical modeling and derivation of analytical expression, and supplementary figures legends". Please view Text S1 here: 
