# Supplementary material for: A Role for the Juxtamembrane Cytoplasm in the Molecular Dynamics of Focal Adhesions
Source: PLoS One. 2009 Jan 28;4(1):e4304. doi: 10.1371/journal.pone.0004304 (PMC2627934; doi:10.1371/journal.pone.0004304)
Supplement: Table S1 — 1st table for the Supporting Information (0.09 MB PDF) [file pone.0004304.s003.pdf]

Table S1. *D* and *b* values derived from fitting the FRAP data from the different locations to the analytical expressions of diffusion, exchange and their combination.

**Location**	**FA (0)**		**Adjacent (1)**		**1.54 μm (2)**		**>5 μm (3)**	
parameter / protein	*D* (μm^2/s)	*b* (s^{-1})	*D* (μm^2/s)	*b* (s^{-1})	*D* (μm^2/s)	*b* (s^{-1})	*D* (μm^2/s)	*b* (s^{-1})
**Paxillin**	0.91	0.11	1.66	1.94	1.75	9.62	2.41	---
**Vinculin**	1.15	0.025	2.68	6.24	3.38	10.12	5.08	---
