# Supplementary material for: Correction: A Role for the Juxtamembrane Cytoplasm in the Molecular Dynamics of Focal Adhesions
Source: PLoS One. 2009 Mar 4;4(3):10.1371/annotation/ce274a43-a284-4839-9791-59e4b2563c9c. doi: 10.1371/annotation/ce274a43-a284-4839-9791-59e4b2563c9c (PMC2665005; doi:10.1371/annotation/ce274a43-a284-4839-9791-59e4b2563c9c)
Supplement: Supplementary file 1 [file pone.ce274a43-a284-4839-9791-59e4b2563c9c.s001.pdf]

Mathematical modeling and derivation of analytical expressions

Section I

Two-dimensional model for diffusion and exchange processes

Our model in section I is based on the following assumptions:

(1) Two-dimensional (2D) diffusion and exchange between the membrane and cytoplasmic proteins (binding to the membrane, detaching, and returning to the cytoplasm). Three dimensions (3D) are discussed in section II.

(2) First-order kinetics and steady-state for the exchanging proteins:

$$k_1 P_m = k_2 P_c \,, \qquad (1)$$

where P_m represents the concentration of proteins in the membrane, and P_c represents the concentration of proteins in the cytoplasm; k_1 and k_2 represent the exchange rates of detaching and binding to the membrane, respectively.

(3) The cytoplasm acts as an infinite reservoir of proteins. Diffusion of the proteins in the cytoplasm is a much faster process than both the diffusion in the membrane and the exchange between the membrane and cytoplasm. The contribution of the cytoplasmic proteins to fluorescence recovery in the membrane is therefore negligible, since after the bleach, which begins each FRAP experiment, the proteins in the cytoplasm quickly diffuse away. As a consequence, Eq. 1 can be rewritten thus:

$$k_1 (P_{mf} + P_{mb}) = k_2 P_c \,, \qquad (2)$$

where P_{mf} represents the concentration of the fluorescent proteins in the membrane, and P_{mb} represents the concentration of the bleached proteins in the membrane.

We used these assumptions to formulate the dynamics of those unbleached membrane proteins that undergo both diffusion and exchange. This dynamic is governed by [1,2]:

$$\frac{\partial P_{mf}}{\partial t} = D\nabla^2 P_{mf} + k_2 P_c - k_1 P_{mf} , \tag{3}$$

which is a diffusion-reaction equation. We considered $k_2 P_c$ to be a constant, since the concentration of fluorescent proteins in the cytoplasm remains approximately constant (the bleached proteins quickly diffuse away). We thus define:

$$h = k_2 P_c \tag{4}$$

and

$$k_1 \equiv b \tag{5}$$

so that Eq. 3 now reads:

$$\frac{\partial P_{mf}}{\partial t} = D\nabla^2 P_{mf} + h - bP_{mf} . \tag{6}$$

Note that the concentrations of the fluorescent membrane proteins are space- and time-dependant: $P_{mf}(r,t)$, while P_c is assumed to be constant.

In FRAP experiments, one measures the fluorescence, which is given by:

$$F(t) = \int_{area} P_{mf}(r,t) I(r) dr^2 , \tag{7}$$

where $I(r)$ is the laser beam intensity. This equation, using the Fourier transform [3] of $P_{mf}(r,t)$, is:

$$F(t) = \int_{area} I(r) dr^2 \int P_{mf}(k,t) e^{-ikr} dk^2 . \tag{8}$$

The second integral in Eq. 8 is the inverse Fourier transform of the concentration of the fluorescent protein. We calculated this concentration using the Fourier transform of Eq. 6:

$$\frac{\partial P_{mf}(k,t)}{\partial t} = h\delta(k) - bP_{mf}(k,t) - Dk^2 P_{mf}(k,t) , \tag{9}$$

which leads to:

$$\log\left[\frac{P_{mf}(k,t)(b+Dk^2)-h\delta(k)}{P_{mf}(k,0)(b+Dk^2)-h\delta(k)}\right]=-(b+Dk^2)t\,, \tag{10}$$

from which we obtain $P_{mf}(k,t)$ required for the fluorescence recovery $F(t)$:

$$P_{mf}(k,t)=\frac{[P_{mf}(k,0)(b+Dk^2)-h\delta(k)]e^{-(b+Dk^2)t}+h\delta(k)}{b+Dk^2}\,. \tag{11}$$

In order to calculate this function, it is necessary to first obtain the behavior of the function at $t=0$, which is the Fourier transform of the initial condition of a FRAP experiment [2].

The Fourier transform of this function constitutes our next step:

$$P_{mf}(k,0)=\int e^{ik\bullet r}P_{mf-0}e^{-\alpha TI(r)}dr^2\,. \tag{12}$$

Where $P_{mf(0)}e^{-\alpha TI(r)}$ is fluorophore concentration profile right after the bleach [2]

When inserting Eq. 12 into Eq. 11 and then into Eq. 8, we finally obtain the fluorescence for the case of both diffusion and exchange:

$$F(t)=R[e^{-bt}F_{ne}(t)+F_0(1-e^{-bt})]+F_0(1-R)\frac{1-e^{-K}}{K}\,, \tag{13}$$

where F_0 represents the fluorescence before the bleach, R the mobile fraction in the membrane, K the bleaching factor, and $F_{ne}(t)$ the fluorescence for the known case of lateral diffusion only [2]:

$$F_{ne}(t)=\sum_{n=0}^{\infty}\frac{(-K)^n}{n!}\frac{1}{(1+n(1+2t/\tau_D))}\,. \tag{14}$$

Two sources contribute to the fluorescence in Eq. 13. The first comes from lateral diffusion fluorescence, which decays exponentially at a time constant that represents the loss of proteins that are exchanged to the cytoplasm. The second comes from proteins originating in the cytoplasm, which bind to the membrane. The total fluorescence at long timescales for the case in which all proteins are mobile (R=1), is due to exchange and equals:

$$F(t \to \infty) = F_0 , \tag{15}$$

which represents the initial fluorescence before the bleach.

Eq. 13 also describes the case when exchange dominates; namely, when the diffusion process occurs at a much slower rate than the exchange process ($\tau_D >> 1/b$). In this instance, Eq. 14 takes the form of the fluorescence at $t = 0$:

$$F(0) = F_0 \frac{1-e^K}{K} , \tag{16}$$

and the corresponding fluorescence is:

$$F(t) = F_0[R(1-e^{-bt}) + (1-R)\frac{1-e^{-K}}{K}] \tag{17}$$

We thus obtained analytical expressions for the extreme case of only exchange, and for the case in which both processes, diffusion and exchange, occur.

Computer simulation of FRAP experiments for proteins moving on a 2D surface

In order to confirm the validity of these expressions, we first performed computer simulations of FRAP experiments. We used a CTRW algorithm [4,5] to simulate proteins capable of moving on a 2D surface by diffusion, exchange, or both. Another condition that had to be taken into consideration was that the exchange process is a steady-state process, meaning that in general, the number of particles leaving the 2D surface must equal the average number of particles reaching this surface. In each case, the simulated data was fitted to the analytical expressions of each model separately (Fig. S2).

Section II

Expansion of the model to a 3D model incorporating FA proteins behavior

We next turned to develop a 3D model of proteins dynamics in FAs. The model we propose divides the total protein population into three subpopulations: one located in the

three-dimensional cytoplasmic volume where proteins perform a free 3D diffusion; a second which performs exchange between the 3D cytoplasmic population and the FA two-dimensional surface, the latter of which serves as the upper boundary of the 3D volume; and an immobile subpopulation located at the FA.

The FRAP behavior of the first population can be described using the expression obtained for the case of lateral diffusion [2] (Eq. 14). Although the diffusion of this subpopulation is a 3D diffusion, rather than a 2D lateral diffusion, Eq. 14 is still valid. The reason is that in a FRAP experiment, the 3D diffusion process is projected onto a 2D surface from which the fluorescence is collected. This is further justified by the fact that although the bleach by the Gaussian beam extends in the z direction, the collection of the fluorescence through the confocal pinhole results in collecting fluorescence from a thin slice in the XY plane. Thus, exchange between molecules that were present on different locations of the z axis during the brief bleach will merely represent replacement of one bleached molecule by another, leaving exchange in the XY plane as the major contributor to the fluorescence recovery measured. These assumptions are supported by the excellent fit of the 3D simulated data (in-silico experiment) to the analytical expression for 2D diffusion (Fig. 3B). The second subpopulation can be described as located in a two-dimensional surface, with its proteins being exchanged with a reservoir of proteins located in the cytoplasmic volume.

The main condition that restricts the usage of this model is that one process must occur on a much shorter timescale, compared to the other process. In the case described herein, the diffusion process should occur much more rapidly than the exchange. In this case, the fluorescence expression is a combination of Eq. 14 and Eq. 17, leading to:

$$F(t) = F_0 R_D \left\{ \sum_{n=0}^{\infty} \frac{(-K)^n}{n!} \frac{1}{1+n(1+2(t/\tau_D))} \right\} + F_0 R_E \left\{ \frac{1-e^{-K}}{K} e^{-bt} + (1-e^{-bt}) \right\} + F_0 (1-R_D-R_E) \frac{1-e^{-K}}{K} \quad (18)$$

Here, R_D is the fraction of proteins performing 3D diffusion, and R_E is the fraction of proteins undergoing exchange.

Figure S1. 2D Simulation of protein dynamics and fitting to analytical expressions for different FRAP mechanisms

Empty circles represent the simulation data, red lines represent the fit to diffusion only, blue lines represent the fit to exchange only, and green lines represent the fit to two populations by diffusion and exchange. (A) Diffusion was simulated with τ = 6666 au (arbitrary units). The simulated diffusion data were well-fitted by diffusion alone (ASD = 0.15) but not by exchange alone (ASD = 2.5). Fitting the analytical expression of two populations by diffusion and exchange did not further improve the fit, despite the additional degree of freedom. Obtaining τ from the fit to diffusion yielded τ = 6702 au. (B) Exchange was simulated with b = $5*10^{-5}$ au^{-1}. The simulated exchange data were well-fitted by exchange alone (ASD = 0.03) but not by diffusion (ASD = 1.5). In this case, too, fitting to the analytical expression of two populations by diffusion and exchange did not improve the fit further (ASD = 0.04). Obtaining b from the fit to exchange yielded $4.97*10^{-5}$ au^{-1}. (C) Simulated recovery which combines diffusion and exchange with τ = 6666 au and b = $2.5*10^{-5}$ au^{-1}. In this case, the simulated data was well-fitted by the expression for the composite recovery (ASD = 0.08) but much less so by each mechanism separately (ASD for diffusion = 1.44; ASD for exchange = 1.98). Obtaining τ and b from the fit to two populations by diffusion and exchange yielded τ = 6633 au and b = $2.4*10^{-5}$ au^{-1}.

Figure S2. FRAP beam-size analysis reveals that the dynamics of paxillin adjacent to FAs are governed by mixed diffusion and exchange

FRAP experiments were conducted on HeLa-JW cells expressing paxillin-YFP (Figure 1D), on a 3 s timescale at areas adjacent to the FAs (locations 1 and 2 in Figure 6). Bars in (A) represent mean ± SEM of 30–40 measurements. In (B), SEM of the τ(63x)/τ(100x) ratios was calculated using bootstrap analysis. Two beam sizes were generated using 40x and 63x, and τ values were determined with each. The ratio between areas illuminated by the two beams is 2.28 ± 0.17 (n=59); this ratio ((B), solid line) is expected for FRAP by lateral diffusion, whereas a ratio of 1 (broken line) is expected for recovery by exchange [6]. (A) τ values derived from FRAP experiments on a short timescale (3 s) at areas immediately adjacent to the FA (left bars) or one beam diameter away from the FA lateral border (right bars). (B) τ(40x)/τ(63x) ratios derived from (A). The τ ratio for the 3 s measurements yielded 1.64 and 1.66 at locations 1 and 2, respectively. This suggests FRAP by a mixed contribution of diffusion and exchange at these locations.

REFERENCES

1. Axelrod D, Koppel DE, Schlessinger J, Elson E, Webb WW (1976) Mobility measurement by analysis of fluorescence photobleaching recovery kinetics. Biophys J 16: 1055-1069.
2. Petersen NO, Felder S, Elson EL (1986) Measurement of lateral diffusion by fluorescence photobleaching recovery. In: Weir DM, Herzenberg LA, Blackwell CC, Herzenberg LA, editors. Handbook of Experimental Immunology. Edinburgh: Blackwell Scientific Publications. pp. 24.21-24.23.
3. Arfken GB (1985) Mathematical methods for physicists. Orlando: Academic Press. xxii, 985 p. p.
4. Montroll EW, Weiss GH (1965) Random Walks on Lattices. II. Journal of Mathematical Physics 10: 167-181.
5. Weiss GH (1994) Aspects and applications of the random walk. Amsterdam ; New York: North-Holland. xiv, 361 p. p.
6. Henis YI, Rotblat B, Kloog Y (2006) FRAP beam-size analysis to measure palmitoylation-dependent membrane association dynamics and microdomain partitioning of Ras proteins. Methods 40: 183-190.
